# Does Ongoing Task Load Influence Prospective Remembering in Autism Spectrum Disorders?

**DOI:** 10.1002/aur.70285

**Published:** 2026-05-29

**Authors:** Daniela Nürnberg, Mareike Altgassen

**Affiliations:** ^1^ Department of Psychology Johannes Gutenberg University Mainz Mainz Germany

**Keywords:** autism, cognitive load, ongoing task, prospective memory

## Abstract

Prospective memory (PM) refers to the cognitive ability to remember to carry out intended actions in the future. The present study investigated PM performance in autistic and non‐autistic adults as well as the impact of the cognitive load of the ongoing task on PM performance. A total of 50 autistic individuals and 51 age‐ and non‐verbal ability‐matched non‐autistic individuals completed an event‐based PM task, which was embedded into an ongoing n‐back task. The cognitive load of the n‐back task was varied (2‐ vs. 3‐back for low versus high cognitive load). Results showed that autistic participants did not differ from non‐autistic participants in their PM performance. The cognitive load of the ongoing task had no impact on event‐based PM performance in both groups. This is the first study to investigate the impact of ongoing task load on event‐based PM performance in autistic adults. The results support the neurodiversity perspective of ASD as a highly heterogeneous population which is not necessarily characterized by reduced PM performance.

## Lay Summary

We investigated prospective memory (PM), the ability to remember what you want to do in the future as well as the impact of the difficulty of the ongoing task (the task that you are doing at the same time as the PM task) on autistic and non‐autistic participants. Autistic participants did not differ from non‐autistic participants in their PM performance. The difficulty of the ongoing task did not influence PM performance. Results demonstrate that autistic individuals have unique characteristics and strengths.

## 1 Introduction

Prospective memory (PM) refers to the cognitive ability to remember and execute intended actions in the future (McDaniel and Einstein 2007). PM is an essential ability for daily living such as remembering to buy groceries from the supermarket or scheduling medical appointments (Landsiedel et al. 2017). Depending on the cue that indicates the appropriate moment to initiate the intended task, PM tasks are defined as either event‐ or time‐based. For event‐based PM tasks a specific stimulus or event indicates the appropriate moment to engage in the intended action (e.g., remember to buy bread when seeing the bakery). For time‐based PM tasks, a specific point in time or an elapsed period of time represents the cue to initiate the PM action (e.g., remember to remove a cake from the oven after 60 min; Kliegel and Jäger 2006). PM is comprised of multiple processes and phases (Kliegel et al. 2011). First, the individual plans an intention for the future and stores it in episodic memory while being engaged in other ongoing tasks. To execute this intention at the appropriate moment, concurrent ongoing activities must be inhibited, and the individual must switch to the prospective action (Sheppard et al. 2018). Hence, PM tasks rely on several cognitive processes, such as attention, episodic memory and executive functions (Sheppard et al. 2018). Importantly, according to the multiprocess framework (McDaniel and Einstein 2000), the involvement of automatic versus strategic executive control processes in the retrieval and execution of PM tasks depends on various factors (e.g., the salience of the PM cue, the importance of the PM task, the nature of the ongoing task or individual differences in cognitive and personality variables). Individuals experiencing difficulties with executive control processes may encounter challenges with PM. For instance, individuals with autism spectrum disorder (ASD), a neurodevelopmental disorder characterized by impaired social communication and interaction, along with restricted, repetitive interests, activities or behaviors (American Psychiatric Association 2013), often show difficulties in executive functions (e.g., inhibition, working memory etc., Craig et al. 2016; Demetriou et al. 2018; Hill 2004; St. John et al. 2022). Moreover challenges with episodic memory (Lind et al. 2014) are reported in autistic individuals.

Indeed, several laboratory studies report reduced event‐ and time‐based PM in autistic individuals compared to non‐autistic individuals (Landsiedel et al. 2017; Sheppard et al. 2018). For example, non‐autistic individuals outperformed autistic participants in event‐ and time‐based PM tasks in the “Dresden Breakfast Task” where participants had to complete various PM tasks by preparing breakfast using real objects, adhering to specific rules and time constraints (Altgassen et al. 2012). Similar results have been reported for the “Virtual Week,” a computerized game which simulates real‐life activities; non‐autistic participants performed superior to autistic adults with regard to event‐ and time‐based PM tasks (Dehnavi and Khan 2024; Kretschmer, Altgassen, et al. 2014).

Importantly, reduced PM performance in ASD seems to occur more consistently in time‐ than event‐based PM tasks. For instance, Landsiedel and Williams (2020) and Williams et al. (2014) asked adult participants to perform an ongoing task of memorizing words and making yes‐or‐no recognition judgments into which time‐ or event‐based PM tasks were embedded. The performance of autistic adults was found to be inferior solely in the time‐based PM task (Landsiedel and Williams 2020; Williams et al. 2014). Consistently, Williams et al. (2013) observed autistic children to exhibit reduced performance in time‐based, but not in event‐based, PM tasks compared to non‐autistic participants in a computer‐based driving game with embedded time‐ and event‐based PM tasks. These findings may be attributed to the fact that time‐based PM tasks put greater demands on executive functions compared to event‐based tasks. In time‐based PM, there is no external cue to prompt retrieval of the intended action, and one must actively monitor and keep in mind the elapsing time (Brandimonte et al. 1996). Research findings on autistic individuals largely report that executive functions underlie PM performance (but see Williams et al. 2013 for a contrasting finding). Reduced event‐based PM performance has been associated with more working memory difficulties in children (Yi et al. 2014) and adults with ASD (Williams et al. 2014). Similarly, Altgassen et al. (2012) reported that enhanced time‐based PM performance was associated with superior switching and working memory performance in autistic adolescents and adults. A subsequent study by Desaunay et al. (2019) argued that the observed difficulties of autistic individuals in the parallel processing of ongoing and PM tasks may be due to their executive functions challenges, specifically the ability to switch between tasks or to inhibit one task.

However, recent studies did not observe difficulties in event‐ and time‐based PM in autism (Faustmann and Altgassen 2024, 2025; Groenman et al. 2024). A potential contributing factor may be that autistic participants in these studies demonstrated high cognitive abilities (e.g., no participants with intellectual disabilities were included) which may have enabled them to compensate for difficulties in executive functions (Faustmann and Altgassen 2024). As postulated by Kliegel et al. (2011) difficulties with PM only occur when task demands exceed individual's cognitive resources. Consequently, it is possible that autistic individuals with high cognitive resources encounter fewer challenges in PM. Moreover, some recent studies used tasks with a rather low cognitive load, finding no differences between autistic and non‐autistic participants in PM performance (Amsterdam Breakfast Task; Groenman et al. 2024). In contrast, reduced PM performance in autistic adults was found in studies employing ongoing tasks with a high cognitive load, for example, the Virtual Week (Dehnavi and Khan 2024; Kretschmer, Altgassen, et al. 2014). According to the multiprocess framework (McDaniel and Einstein 2000), the complexity of the ongoing task can influence PM performance (e.g., low vs. high demands on executive functions). To illustrate, Rendell et al. (2007) discovered that event‐based PM challenges in older adults disappeared when the complexity of the ongoing task was reduced. Concordantly, Meier and Zimmermann (2015) showed that the cognitive load of an ongoing task affected remembering to execute the PM task in young adults. Therefore, an important question is whether the frequently observed reduced PM performance in autistic individuals, relative to non‐autistic participants, can be attributed to the cognitive demands of the ongoing task exceeding the cognitive resources of autistic participants. To date, no studies have been conducted that have investigated the impact of the cognitive load of the ongoing task on PM performance in adults with and without autism. Research into the manipulation of the cognitive load of the PM task has indicated that comparison and ASD participants perform better under PM tasks with low compared to high retrospective memory load (Henry et al. 2014; Kretschmer, Altgassen, et al. 2014). A further study without PM found that autistic children demonstrated a higher number of errors on a working memory n‐back task compared to non‐autistic participants, as the cognitive load increased (de Vries and Geurts 2014).

The objective of the present study was to examine PM in autistic adults in comparison to non‐autistic adults and to investigate the impact of the cognitive load of a n‐back task (ongoing task) on PM performance. The 2‐ and 3‐back tasks were specifically selected to manipulate cognitive load, based on their successful utilization in prior related studies with autistic and non‐autistic adults (Nyberg et al. 2009; van Steenburgh et al. 2017). It is important to investigate these potential associations, as the findings could have practical implications for autistic individuals, for example, by avoiding multi‐tasking or by adding reminders to reduce their cognitive load and improve their PM in day‐to‐day life. Given the increasing heterogeneity within the autism spectrum, it is essential to examine large samples to obtain meaningful data (Lombardo et al. 2019). Consequently, an online study format was selected to facilitate the efficient recruitment of many autistic individuals worldwide. Online platforms are an important tool for reaching a more extensive and heterogeneous group of autistic adults, including those unable to participate in laboratory studies. However, this approach introduces unique methodological challenges, particularly concerning internal and external validity due to the heavy reliance on participant self‐report (Rubenstein and Furnier 2021). Concurrently, online testing has the potential to mitigate social pressures, social desirability, and anxiety. This could result in autistic individuals feeling less observed and stressed, potentially positively impact their performance in test situations (Do et al. 2025).

In line with previous research (Altgassen et al. 2012; Dehnavi and Khan 2024; Kretschmer, Altgassen, et al. 2014), we expected adults with ASD to show reduced PM performance as compared to age‐and ability‐matched non‐autistic adults. Based on the results of the manipulation of the cognitive load of the PM task, we expected both groups to show better PM performance under low compared to high cognitive load of the ongoing task (Henry et al. 2014; Kretschmer, Altgassen, et al. 2014). Furthermore, we hypothesized that individuals with ASD exhibit greater challenges in PM performance under high versus low cognitive demands of an ongoing working memory n‐back task in comparison to non‐autistic individuals (de Vries and Geurts 2014).

## 2 Methods

### 2.1 Participants

Fifty‐nine non‐autistic and 79 autistic participants were recruited from the online platform *Prolific* in the United Kingdom, the United States, Australia and Canada. As this study is among the first to utilize the recruitment platform Prolific with autistic participants, the sample size was guided by comparable studies with autistic participants using online recruitment or online data collection (Belcher et al. 2022; Westerberg et al. 2023). Eight non‐autistic and 29 autistic participants had to be excluded; mainly due to not meeting (autistic individuals) or surpassing (comparison group) the Comprehensive Autistic Trait Inventory (CATI) cut‐off or severe co‐occurring psychiatric or neurological disorders (see exclusion criteria). Furthermore, data sets were excluded when participants appeared to have not completed all tasks thoroughly and conscientiously, or failed the previously specified quality checks (e.g., zero correct Go trials in the Go‐NoGo task, minimal time spent on a task or questionnaire). The final study sample comprised a total of 101 adults, including 50 autistic participants (27 males, 23 females) and 51 non‐autistic participants (27 males, 24 females). To achieve a balanced gender ratio between the groups, non‐autistic participants were recruited using targeted pre‐screening criteria to match the exact gender distribution of the previously recruited autistic group. Participant characteristics are presented in Table 1. The inclusion criteria specified that participants had to be aged between 18 and 65 years, be native English speakers, and, for those in the ASD group, have received a clinical diagnosis of ASD. Of the autistic participants, 48% were diagnosed during childhood, while 52% were diagnosed during adulthood. To confirm the certainty of the self‐reported ASD diagnosis, the CATI was used as an objective measure. All non‐autistic participants with a CATI total score above 152 (the upper limit of the 95% confidence interval for the cut‐off threshold for the classification of autism, which made a clinical diagnosis of autism highly probable) were excluded, as were all autistic participants who were below the 95% confidence interval (i.e., under 136; English et al. 2024). Further exclusion criteria were severe psychiatric or neurological disorders (e.g., schizophrenia, bipolar disorder or Parkinson's disease) and psychotropic substance use. The comparison group was matched to the autism group on the following criteria: chronological age, gender, educational qualifications and non‐verbal ability on the matrices reasoning subtests of the Wechsler Adult Intelligence Scale‐Fourth Edition (WAIS‐IV). There were no significant differences regarding these variables between the groups (see Table 1). However, as anticipated, the groups differed in the self‐reported autistic traits (CATI total score) with autistic participants reporting significantly higher scores. Among the 50 autistic participants, 4% had obtained the educational qualification of secondary education/General Certificate of Secondary Education (GCSE), 20% held A‐levels/high school qualifications, 42% had obtained a bachelor's degree, 24% had obtained a master's degree, and 10% held a PhD or professorship. For the comparison group, 3.9% had a secondary education/GCSE, 15.7% had A‐levels/high school qualifications, 41.2% had a bachelor's degree, 27.5% had a master's degree, and 11.8% had a PhD or professorship. In the ASD group, 56% indicated the presence of a co‐occurring psychological disorder, with affective and anxiety disorders being the most prevalent. In the comparison group, 5.9% reported a psychological disorder (depression and anxiety disorder). The study was conducted in accordance with the Declaration of Helsinki and reviewed by the local university ethics committee. All participants provided online written informed consent. Participants were compensated for their involvement in the study with the recommended payment (9 pounds per hour) provided by Prolific.

**TABLE 1:** Participants characteristics.

	Autistic (*n* = 50) *M* (SD); range	Non‐autistic (*n* = 51) *M* (SD); range	*F* (df)	*η* ^2^
Age	36.0 (10.9); 19–62	36.7 (11.0); 20–64	0.094 (1,99)	0.001
NVA age‐scaled scores	6.9 (3.1); 2–13	7.2 (2.9); 2–12	0.131 (1,99)	0.001
CATI total score	169.3 (14.8); 141–200	108.7 (27.7); 52–151	186.773 (1,99)***	0.654

*Note:* ***p < 0.001.

Abbreviation: NVA, non‐verbal ability.

### 2.2 Materials

All questionnaires and tests were created and collected online using the experiment builder *Gorilla* and the online recruitment platform *Prolific*.

#### 2.2.1 Non‐Verbal Abilities

The matrices reasoning subtest of the German version of the WAIS‐IV (Wechsler 1955) was utilized to assess participants' non‐verbal abilities. Participants were invited to identify patterns within a given design. As the task progressed, the level of difficulty increased with each matrix. Subsequently, raw scores were converted into age‐scaled scores. Higher scores indicate superior performance.

#### 2.2.2 Autism Severity

The severity of autism was measured using the CATI (English et al. 2021). The CATI is a self‐report questionnaire measuring autistic traits in autistic and non‐autistic adults. The CATI is composed of 42 items, which are divided into six subscales. Each of these subscales comprises seven items, with the specific subscales being *Social Interactions*, *Communication*, *Social Camouflage*, *Repetitive Behaviors*, *Cognitive Rigidity*, and *Sensory Sensitivity*. The items are scored on a 5‐point Likert‐scale ranging from “definitely disagree” to “definitely agree.” The total score ranges from 42 to 210 points. A higher score is indicative of higher autistic traits. The optimal cut‐off threshold for the classification of autism is 148 (95% confidence interval 136–152; English et al. 2024).

#### 2.2.3 Executive Function Inhibition

The assessment of inhibition was conducted utilizing the “Go‐NoGo” test (Donders 1969; Gomez et al. 2007). Participants were instructed to press the space bar in response to the presentation of a “star” symbol and to refrain from responding to the presentation of a “heart” symbol. Items were presented for 1000 ms with an interstimulus interval of 500 ms between trials. Twenty‐five percent of all stimuli were NoGo trials. Participants completed a practice block with feedback consisting of 24 trials. This was followed by 180 task trials. In addition to the correct pressing of the space bar, the reaction speed was measured for Go trials. The more accurate the performance of the task, the better the overall performance. Dependent variables were the accuracy (number of correct responses) and the response time of the Go trials and the accurate inhibition (number of correct responses) of the NoGo trials.

#### 2.2.4 Executive Function Working Memory

**FIGURE 1:**
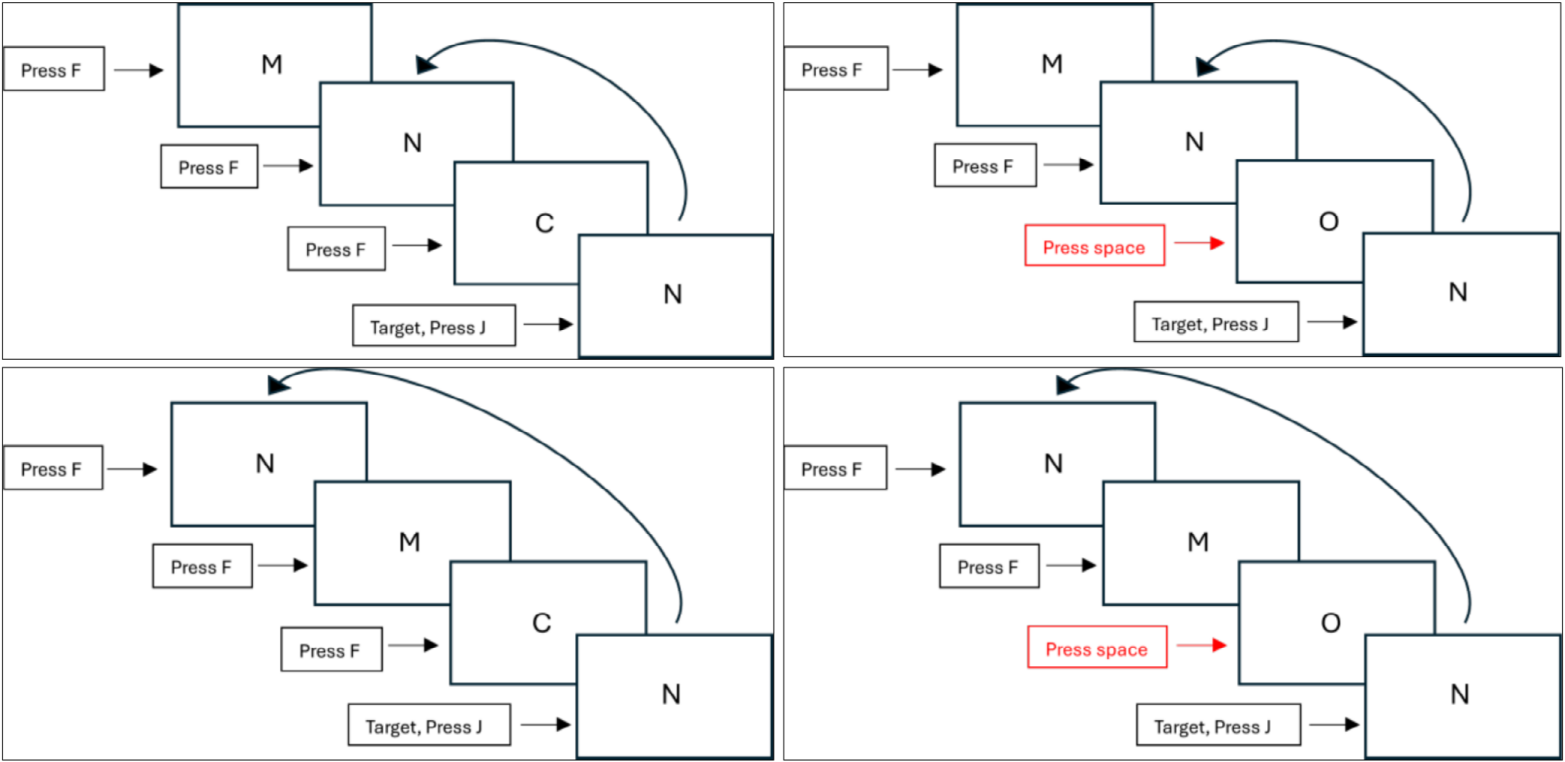
Single and dual n‐back tasks with and without the PM task. The single 2‐back task is shown at the top left, the dual 2‐back task with the PM task is shown at the top right. The single 3‐back task is shown at the bottom left, the dual 3‐back task with the PM task is shown at the bottom right.

The “n‐back task” (Kirchner 1958) was concurrently used as an ongoing task for the PM task and the single task block to measure working memory performance. The 2‐ and 3‐back tasks were chosen as they are well‐established in current literature to operationalize varying levels of cognitive load (Nyberg et al. 2009; van Steenburgh et al. 2017). Letters (except for vowels) were displayed one by one on a computer screen. In the 2‐back conditions (low cognitive load), participants were required to press the J‐key if the letter they saw on‐screen corresponded to one they saw two letters before. For all the other letters, the F‐key was utilized (see Figure 1). In the 3‐back conditions (high cognitive load), the task was identical, with the exception that participants were required to press the J‐key when the letter they saw on‐screen corresponded to one they saw three letters before. Items were presented for 1500 ms with an interstimulus interval of 500 ms between trials. Twenty‐five percent of all the stimuli presented were target items. Participants were first asked to complete a practice block with feedback comprising 24 trials. This was followed by 40 task trials (a single task block), after which the PM task was introduced (see PM task). All participants completed both conditions (2‐ and 3‐back) of the experiment, which were randomized in order. To control for order effects, the presentation of these conditions was counter‐balanced for all participants. Dependent variables were the accuracy (number of correct responses) and the reaction time of the responses.

#### 2.2.5 Prospective Memory

PM performance was assessed with an event‐based PM task during the 2‐ and 3‐back task (“dual task”) based on the study by Altgassen et al. (2021). Following the completion of the single 2‐ or 3‐back task, participants were informed of an additional requirement when performing the task again, namely, to press the space bar whenever a vowel was displayed on the screen (A, E, I, O, U; see Figure 1). Following the instruction and practice with 16 items (2 PM cues and 14 ongoing trials) with feedback on this dual task, participants were instructed to memorize this task. Participants were required to perform the CATI or the matrices test to ensure a filled delay between the PM instructions and the PM task. Subsequently, participants had to perform the dual task that they had previously practiced. The dual task block comprised 120 trials with 6 PM hits (vowels) in total. Dependent variables were the accuracy (number of correct responses) and the reaction time of the PM and ongoing task responses.

#### 2.2.6 Cognitive Load

The perceived cognitive load of the dual 2‐ and 3‐back task was assessed using the PAAS‐Scale (Paas 1992). The PAAS‐Scale consists of a 9‐point Likert‐Scale ranging from 1 = “very, very low mental effort” to 9 = “very, very high mental effort.”

### 2.3 Procedure

First, after reading the study information, participants had to agree online to the declaration of consent. Participants completed the socio‐demographic questionnaire. Following the single 2‐ or 3‐back task (start condition was randomized), the event‐based PM task was introduced and practiced. The CATI screening questionnaire was completed as a filled delay between the instruction and performance of the PM task. This was followed by a dual task block, comprising the n‐back and PM task. The subjective cognitive load of the dual task was subsequently measured using the PAAS scale. The same procedure was repeated for the remaining condition of the n‐back task (2‐ or 3‐back). First, participants practiced the remaining single condition of the n‐back task and subsequently the event‐based PM task was introduced (dual task) and practiced. The WAIS‐IV matrices test was administered to ensure a filled delay between the instruction and performance of the PM task. Following this, the dual task block was performed, and then its subjective cognitive load was assessed. Finally, the Go No‐Go test was performed.

## 3 Results

The descriptive data for the ongoing and PM tasks is set out in Table 2. To account for the broad age range of the participants, all analyses were repeated using analyses of covariance (ANCOVA) with age as a covariate. These supplementary analyses yielded consistent results, confirming that chronological age did not systematically bias the findings. To explore potential gender effects, all main analyses (PM, cognitive load and ongoing task) were repeated with gender as an additional between‐subjects factor, and gender did not systematically influence the pattern of results for the main dependent variables.

**TABLE 2:** Mean and standard deviations of performance in PM, executive functions and self‐reported cognitive load.

	Autistic (*n* = 50); *M* (SD)	Non‐autistic (*n* = 51)*; M* (SD)
PM performance		
Dual 2‐back accuracy	0.464 (0.317)	0.454 (0.325)
Dual 3‐back accuracy	0.528 (0.304)	0.494 (0.333)
Dual 2‐back response times	0.914 (0.210)	0.925 (0.203)
Dual 3‐back response times	0.963 (0.228)	0.919 (0.233)
Ongoing Task		
Single 2‐back accuracy (WM)	0.843 (0.141)	0.843 (0.167)
Single 3‐back accuracy (WM)	0.723 (0.183)	0.778 (0.147)
Dual 2‐back accuracy	0.819 (0.187)	0.843 (0.159)
Dual 3‐back accuracy	0.728 (0.166)	0.752 (0.172)
Single 2‐back response times (WM)	0.639 (0.160)	0.670 (0.169)
Single 3‐back response times (WM)	0.712 (0.196)	0.677 (0.154)
Dual 2‐back response times	0.726 (0.163)	0.740 (0.128)
Dual 3‐back response times	0.777 (0.161)	0.760 (0.149)
Inhibition		
Go trials accuracy	0.980 (0.048)	0.987 (0.024)
NoGo trials accuracy	0.878 (0.090)	0.860 (0.117)
Go trials response times	0.426 (0.092)	0.420 (0.067)
Self‐reported cognitive load		
PAAS 2‐back	7.56 (1.13)	7.02 (1.45)
PAAS 3‐back	7.80 (1.4)	7.43 (1.15)

*Note:* Response times, reaction speed in seconds based on correct trials only; accuracy, performance, ranging from 0 to 1, with 1 representing the best possible performance.

Abbreviations: PM, prospective memory; WM, working memory.

### 3.1 PM Performance and Cognitive Load

#### 3.1.1 PM Accuracy and Response Time

To analyze the impact of the cognitive load of the two dual task conditions (dual 2‐back vs. dual 3‐back) and group status (autistic vs. non‐autistic) on PM performance (accuracy), a 2 × 2 mixed analysis of variance (ANOVA) was conducted. There was no significant interaction between group status and task conditions regarding accuracy, *F* < 1. There was no significant main effect of task condition, *F* (1,99) = 2.97, *p* = 0.087, *η*_*p*_^2^ = 0.029, indicating no differences in PM hits between the task conditions. There was also no significant main effect of group status, *F* < 1, revealing no differences in PM hits between autistic and non‐autistic participants.

To analyze the impact of the cognitive load of the two dual task conditions (dual 2‐back vs. dual 3‐back) and group status (autistic vs. non‐autistic) on PM response times, a 2 × 2 mixed ANOVA was conducted. There was no significant interaction between group status and task conditions regarding response time, *F* (1,77) = 1.18, *p* = 0.280, *η*_*p*_^2^ = 0.015, and no significant main effect of task condition, *F* < 1, suggesting no differences in the response time of the PM task between the task conditions. There was no significant main effect of group status, *F* < 1, indicating no differences in response times between the groups.

#### 3.1.2 Ongoing Task

A 2 × 2 × 2 mixed ANOVA was conducted to analyze the impact of task block (single vs. dual), task load (2‐back vs. 3‐back), and group status (autistic vs. non‐autistic) on ongoing task accuracy. None of the two‐way interactions reached significance, *F* < 1. The three‐way interaction was also not significant, *F* (1, 99) = 3.153, *p* = 0.079, *η*_*p*_^2^ = 0.031. There was a significant main effect of task load, *F* (1, 99) = 38.405, *p* < 0.001, *η*_*p*_^2^ = 0.279, with both groups performing better on the 2‐back compared to the 3‐back conditions. There was no significant main effect of task block, *F* (1, 99) = 1.528, *p* = 0.219, *η*_*p*_^2^ = 0.015, and group status, *F* < 1, revealing that both groups showed a similar performance in ongoing task accuracy.

A 2 × 2 × 2 mixed ANOVA was conducted to analyze the impact of task block (single vs. dual), task load (2‐back vs. 3‐back), and group status (autistic vs. non‐autistic) on ongoing task response times. A significant task load and group interaction was found, *F* (1, 99) = 5.456, *p* = 0.022, *η*_*p*_^2^ = 0.052, indicating that the increase in response times from the 2‐back to the 3‐back condition was more pronounced in the autistic group than in the non‐autistic group. No other two‐way or three‐way interactions reached significance, *F* < 1. There was a significant main effect of task block, *F* (1, 99) = 51.854, *p* < 0.001, *η*_*p*_^2^ = 0.344, with longer response times in the dual‐task conditions compared to the single‐task conditions, and a significant main effect of task load, *F* (1, 99) = 13.097, *p* < 0.001, *η*_*p*_^2^ = 0.117, with longer response times in the 3‐back compared to the 2‐back conditions. There was no significant main effect of group status, *F* < 1, indicating that both groups showed a similar performance in ongoing task response time.

#### 3.1.3 Subjective Cognitive Load

To analyze the impact of the cognitive load of the dual task conditions (dual 2‐back vs. dual 3‐back) and group status (autistic vs. non‐autistic) on the self‐reported cognitive load (PAAS‐Scale), a 2 × 2 mixed ANOVA was conducted. There was no significant interaction between group status and task conditions regarding the self‐reported cognitive load, *F* < 1. There was a significant main effect of task condition, *F* (1,99) = 7.10, *p* = 0.009, *η*_*p*_^2^ = 0.067, suggesting a higher self‐reported cognitive load in the dual 3‐back compared to the dual 2‐back condition of both groups. There was a significant main effect of group status, *F* (1,99) = 4.05, *p* = 0.047, *η*_*p*_^2^ = 0.039, with autistic participants reporting a higher cognitive load compared to non‐autistic participants in both task conditions.

### 3.2 Executive Functions

#### 3.2.1 Working Memory

See ongoing task results.

#### 3.2.2 Inhibition

Univariate ANOVAs were conducted to analyze the inhibition performance between the groups. No significant group differences were observed in the accuracy of the Go Stimuli, *F* < 1, in the accuracy of the NoGo Stimuli (inhibition), *F* < 1, nor in the response time of the Go‐Stimuli, *F* < 1.

### 3.3 PM and Executive Functions

Correlational analyses (Pearson) were conducted to explore the relationships between performance in PM and executive functions (see Tables 3 and 4). Fisher's *r*‐to‐*z* transformations were used to compare the strength of correlations between prospective memory and executive functions across autistic and non‐autistic participants. The correlation between PM in the dual 2‐back condition and working memory (3‐back), which was significant in the non‐autistic group (*r* = 0.289, *p* = 0.040) but not in the autistic group (*r* = 0.078, *p* = 0.591), did not differ significantly between groups, *Z* = 1.07, *p* = 0.285.

**TABLE 3:** Pearson correlations non‐autistic participants.

	PM hits dual 2‐back	PM hits dual 3‐back	Inhibition	WM 2‐back
PM hits dual 3‐back	0.638**			
Inhibition	0.053	0.125		
Single 2‐back (WM)	0.163	0.141	0.409**	
Single 3‐back (WM)	0.289*	0.253	0.311*	0.453**

*Note:* *p < 0.05; **p < 0.01.

Abbreviation: WM, working memory.

**TABLE 4:** Pearson correlations of autistic participants.

	PM hits dual 2‐back	PM hits dual 3‐back	Inhibition	WM 2‐back
PM hits dual 3‐back	0.472**			
Inhibition	0.041	0.119		
Single 2‐back (WM)	0.147	0.223	0.116	
Single 3‐back (WM)	0.078	0.145	−0.210	0.358*

*Note:* *p < 0.05; **p < 0.01.

Abbreviation: WM, working memory.

## 4 Discussion

### 4.1 PM Performance and Cognitive Load

The objective of this study was to investigate PM performance in adult participants with and without autism and to explore the impact of the cognitive load of the ongoing task on event‐based PM performance.

In contrast to our expectations, we observed no significant differences in event‐based PM performance between autistic and non‐autistic adults. These results contradict the extant literature examining adults with ASD (Altgassen et al. 2012; Kretschmer, Altgassen, et al. 2014), yet are in accordance with the study by Williams et al. (2014) where difficulties in PM were found in time‐based but not in event‐based PM in autistic adults. Moreover, these findings are consistent with more recent studies that observed no PM difficulties in autistic compared to non‐autistic adults (Faustmann and Altgassen 2024, 2025; Groenman et al. 2024). The absence of group differences in recent studies may be attributable to the increasing heterogeneity of individuals' characteristics' within the autistic spectrum (Lombardo et al. 2019). The heightened public awareness of ASD and the broadening of diagnostic criteria for autism are leading to an increased number of individuals being diagnosed with ASD (Hull et al. 2020; Zeidan et al. 2022) and to more and more subgroups of autistic individuals (Mottron and Bzdok 2020). This development presents a potential opportunity for individuals with milder autistic characteristics—as compared to earlier diagnosed individuals—to participate in research studies which may influence their outcomes (Rødgaard et al. 2019). Furthermore, in the studies conducted by Faustmann and Altgassen (2024, 2025) the proportion of female participants exceeded that of male participants, despite the fact that autism is more frequently diagnosed in males than females and autistic women are underdiagnosed (Rynkiewicz et al. 2019). Given that autism may manifest differently in women than in men (so‐called female autism phenotype; Hull et al. 2020), the higher proportion of females as compared to males may have influenced study findings (Faustmann and Altgassen 2024, 2025). The present study demonstrates a balanced gender ratio in contrast to older autism studies (Farrant et al. 1999; Mackinlay et al. 2006), a factor that could have influenced results (Frazier et al. 2014; Hervas 2022; Hull et al. 2020). Furthermore, like the present study, the studies by Faustmann and Altgassen (2024, 2025) were conducted exclusively via online platforms which may have impacted on the results, given that during testing, social pressure, social desirability and anxiety may be diminished (Do et al. 2025). This could potentially result in autistic individuals feeling less observed and stressed, making them less self‐conscious, which may in turn enhance their performance in test situations. Furthermore, the composition of the sample may vary as compared to standard lab‐based studies, with individuals who would not participate in person potentially doing so online.

Regarding the cognitive load of the ongoing task, we found no significant impact on PM performance in either group. This finding contrasts with the results of earlier studies investigating the cognitive load of the PM task (Henry et al. 2014; Kretschmer, Altgassen, et al. 2014). Furthermore, in contrast to our expectations, individuals with ASD demonstrated similar PM performance compared to non‐autistic participants under high versus low cognitive demands of the ongoing task. However, there was a non‐significant tendency that both groups demonstrated enhanced PM performance in the dual 3‐back relative to the dual 2‐back task, although the subjective cognitive load (PAAS scale) was found to be higher in the dual 3‐back compared to the dual 2‐back task. It is possible that with higher cognitive load of the ongoing task, participants' focus may have shifted to the simpler PM task. These observations are in line with the results of Desaunay et al. (2019). Here, individuals with ASD showed difficulties in processing parallel ongoing and PM tasks, though not in performing the ongoing task alone. Consequently, the challenge for autistic individuals appears to be having to maintain focus on two tasks concurrently, thereby enabling the prioritization of one task over another. Possibly, a slight to moderate increase in the cognitive load of the ongoing task may cause PM performance to decline. However, if the cognitive load of the ongoing task becomes too high, PM performance may improve as participants divert their attention away from the challenging ongoing task to the less complex PM task. To test this, future studies should manipulate the cognitive load of the ongoing task in smaller steps (e.g., at least 3 levels of cognitive load for the ongoing task) and examine its impact on PM performance.

Regarding the self‐reported cognitive load, autistic participants rated their subjectively perceived cognitive load of the dual 2‐ and 3‐back conditions significantly higher than non‐autistic participants, although their performance was comparable. This discrepancy may be due to autistic individuals having elevated expectations of the demands of a given task, or having negative self‐perceptions of their capabilities, which may result in an underestimation of their true abilities (Huggins et al. 2021). However, it is also possible that the dual tasks were more challenging for autistic participants, requiring them to exert more cognitive effort to achieve the same level of performance as the non‐autistic participants.

### 4.2 PM and Executive Functions

With respect to executive functions, we found no significant differences in working memory and inhibition performance between autistic and non‐autistic adults in contrast to the extant literature (Demetriou et al. 2018; Hemmers et al. 2022; St. John et al. 2022; Xie et al. 2020). However, contrasting findings have also been reported (Abbott et al. 2018; Geurts et al. 2014; Kleinhans et al. 2005; Kretschmer, Lampmann, and Altgassen 2014) where no significant executive function differences between autistic and non‐autistic adults were found. The observed differences in findings may be explained by the heterogeneity of the autistic spectrum which may be reflected in the results, given that small samples and different subgroups of autistic individuals were often examined across studies (Geurts et al. 2014). For instance, the age at which an individual is diagnosed with autism may have a significant impact on the outcome. Research indicates that individuals diagnosed with autism in adulthood exhibit less challenges with executive functions when compared to those diagnosed during childhood or adolescence (Abbott et al. 2018). In the present study, almost half of the autistic individuals were not diagnosed until adulthood which could have influenced the outcomes. Moreover, our results underline that autistic individuals can have full functionality in some executive functions, highlighting the importance of considering neurodiversity and recognizing autistic individuals' strengths and diverse cognitive profiles (Pellicano and den Houting 2022).

Concerning the relationship between executive functions and PM performance, we found in the comparison group that better ongoing task performance (working memory) in the single 3‐back condition was associated with better event‐based PM performance in the dual 2‐back condition. In contrast, we found no relationship between performance in event‐based PM and executive functions in the autistic group, and Fisher's *r*‐to‐*z* transformations showed that the groups did not differ significantly, *Z* = 1.07, *p* = 0.285. Moreover, no relationship between inhibition and event‐based PM performance was found in both groups. The absence of certain associations could be attributed to the type of PM task, as Altgassen et al. (2012) found an association between executive functions and PM only for time‐based, but not for event‐based PM tasks. This observation is consistent with the established finding that time‐based PM tasks put greater demands on executive function than event‐based PM tasks (Brandimonte et al. 1996) and relations may be therefore stronger for executive functions and time‐ compared to event‐based PM. Furthermore, the absence of substantial correlations can be attributed to the minimal variability observed in executive function performance, given that both groups exhibited comparable executive functioning. Consequently, the results of this study suggest that inhibition and working memory do not appear to play a significant role in the event‐based PM performance of adults with and without ASD.

### 4.3 Limitations and Future Research

It is important to acknowledge the general limitations of online studies. The capacity to verify to what extent the participants were performing to their full potential was limited, and online data is more prone to errors. Additionally, individuals lacking computer access or digital literacy may be systematically excluded, which could limit the generalizability of the findings. Consequently, data sets of questionable quality were excluded, and the self‐reported autism diagnoses were supplemented with the CATI total score. At the same time, online studies have the capacity to reach a more extensive and heterogeneous group of autistic participants worldwide, including those who are unable to participate in laboratory studies due to mobility issues or social anxiety. A further limitation concerns the use of the CATI, which relies on self‐reported diagnoses without clinician confirmation. While the CATI is a robust measure of autistic traits, it cannot replace clinician‐administered diagnostic assessments, and the findings must be interpreted within the context of this limitation. Further research could investigate possible differences in the outcomes of online and face‐to‐face testing, and the factors that are associated with these potential variations. Furthermore, research could be conducted into the cognitive load and parallel task processing in autistic individuals, with a view to deriving therapeutic approaches.

## 5 Conclusion

In summary, we found no performance differences between autistic and non‐autistic participants in an event‐based PM task. The cognitive load of the ongoing task had no impact on PM performance in both groups. The results support the neurodiversity perspective that ASD is heterogeneous and not necessarily characterized by reduced PM performance.

## Funding

This work was supported by Stiftung Irene.

## Conflicts of Interest

The authors declare no conflicts of interest.

## Data Availability

The data that support the findings of this study are available from the corresponding author upon reasonable request.
